# Effects of Electroacupuncture of Different Frequencies on the Release Profile of Endogenous Opioid Peptides in the Central Nerve System of Goats

**DOI:** 10.1155/2012/476457

**Published:** 2012-10-24

**Authors:** Li-Li Cheng, Ming-Xing Ding, Cheng Xiong, Min-Yan Zhou, Zheng-Ying Qiu, Qiong Wang

**Affiliations:** College of Veterinary Medicine, Huazhong Agricultural University, Wuhan 430070, China

## Abstract

To investigate the release profile of met-enkephalin, **β**-endorphin, and dynorphin-A in ruminants' CNS, goats were stimulated by electroacupuncture of 0, 2, 40, 60, 80, or 100 Hz for 30 min. The pain threshold was measured using potassium iontophoresis. The peptide levels were determined with SABC immunohistochemisty. The results showed that 60 Hz increased pain threshold by 91%; its increasing rate was higher (*P* < 0.01) than any other frequency did. 2 Hz and 100 Hz increased met-enkephalin immunoactivities (*P* < 0.05) in nucleus accumbens, septal area, caudate nucleus, amygdala, paraventricular nucleus of hypothalamus, periaqueductal gray, dorsal raphe nucleus, and locus ceruleus. The two frequencies elicited **β**-endorphin release (*P* < 0.05) in nucleus accumbens, septal area, supraoptic nucleus, ventromedial nucleus of hypothalamus, periaqueductal gray, dorsal raphe nucleus, locus ceruleus, solitary nucleus and amygdala. 60 Hz increased (*P* < 0.05) met-enkephalin or **β**-endorphin immunoactivities in the nuclei and areas mentioned above, and habenular nucleus, substantia nigra, parabrachial nucleus, and nucleus raphe magnus. High frequencies increased dynorphin-A release (*P* < 0.05) in spinal cord dorsal horn and most analgesia-related nuclei. It suggested that 60 Hz induced the simultaneous release of the three peptides in extensive analgesia-related nuclei and areas of the CNS, which may be contributive to optimal analgesic effects and species variation.

## 1. Introduction

Acupuncture is a traditional therapeutic technique in Oriental medicine, which has a long history of 4000 years. As a modern version of hand acupuncture, electroacupuncture (EA) can provide a valid analgesic effect and has little physiological interference [1, 2]. It was successfully used to ameliorate pain not only in varieties of painful diseases [3, 4], but in various operations, such as cesarean section, gastrectomy, enterectomy, and castration, in animals during the 1970s [1, 5]. Since then, analgesia-regulating mechanism of EA has been extensively investigated. Previous studies found that electroacupuncture analgesia (EAA) was involved in modulations of neurotransmitters or neuromodulators in the central nerve system (CNS) [6], and most early studies focused on the role of neurotransmitters such as serotonin, noradrenaline, dopamine, and acetylcholine. Later, it was certified that some endogenous opioid peptides (EOPs), mainly including enkephalin, *β*-endorphin, and dynorphin, played a more important role in EAA [7, 8]. EA of different frequencies can promote the release of different EOPs in the CNS. Studies showed that EAA induced by 2 Hz (low frequency) was mediated by the release of met-enkephalin (M-ENK) and *β*-endorphin (*β*-EP), while EAA by 100 Hz (high frequency) was mediated by the release of dynorphin-A (DYN-A) in the CNS in rats [9–11]. Although these results in rats above are extrapolated to give reasonable explanations for acupuncture analgesia phenomenon and its treatment of related diseases in human, there are still some unknown mechanisms to be investigated.

It has been proved that analgesia induced by EA varies in animal species. In order to quantitatively estimate the degree of acupuncture-induced analgesia, some researchers used an anesthetic to ensure a complete analgesia and to assess the reduction of the amount of the anesthetics consumed in the EA plus anesthetic group as compared to the anesthetic group without acupuncture. Studies showed that EA in combination with anesthetics resulted in the reduction of the dosage of the anesthetics in human, rat, and goat by 45%–55%, 50%–60%, and over 75%, respectively [12, 13]. It is clear that the analgesic effect induced by EA in goats (ruminants) is superior to that in rats or human. Therefore, the modalities of EOP release elicited by different frequencies in ruminants could be different from those in rats. In the present study, goats were stimulated with EA of different frequencies to determine the analgesic efficacy and the release levels of M-ENK, *β*-EP, and DYN-A in the CNS in order to probe into the mechanisms of EA-induced analgesia in ruminants.

## 2. Materials and Methods

### 2.1. Animal Preparation

Forty-nine healthy 1- to 2-year-old hybrid male goats, weighing 23–28 kg, were purchased from the goat farm of Hubei Agricultural Academy of Science. All experimental goats were randomly divided into seven groups of seven each, maintained on dry grass diet which was supplemented with a cereal-based concentrate, and drank freely. They were dewormed and accustomed to being approached. Feed was withheld for 24 h before the start of the experiment. The experiment was performed in a quiet environment, and the ambient temperature fluctuated between 23°C and 24°C. The experimental protocol was approved by the Animal Care Center, College of Veterinary Medicine, Huazhong Agricultural University, Wuhan, China.

### 2.2. Electroacupuncture

A set of Baihui (hundred meetings), Santai (three platforms), Ergen (ear base), and Sanyangluo (three Yang communications) points was selected for EA. The anatomic location of these points has been described in detail in veterinary medicine [12, 14]. Needle insertion and EA were conducted with the method reported by Liu et al. [12]. Experimental animals were restrained in right recumbency, and stimulated with EA at 0, 2, 40, 60, 80, or 100 Hz for 30 min via WQ-6F Electronic Acupunctoscope (Beijing Xindonghua Electronic Instrument Co., Ltd., Beijing, China). The goats which were only dealt with needles left in the acupoints without electricity were used as the sham control.

### 2.3. Determination of Pain Threshold

Just before and after EA, the pain threshold was measured on the center of the left flank using the method of potassium iontophoresis [12, 15, 16]. The region used to measure pain threshold was shaved, cleaned with soap and water, and sterilized with 75% alcohol. Two electrodes soaked with saturated potassium chloride were placed 3 cm apart on the skin in position. A galvanofaradism apparatus (Shantou Medical Equipment Factory Co., Ltd., Shantou, China) was used to deliver pulsed direct current to the electrodes. The voltage was increased stepwise. Obvious contraction of the local skin and muscle was taken as the endpoint; the current was then terminated, and the volt level was recorded. The procedure was repeated three times. The average volt level was obtained. Mean voltages before and after EA were expressed as Vo and Vn, respectively. The change of percentage in pain threshold was calculated as follows: ∆(%) = (Vn–Vo)/Vo × 100%.

### 2.4. Measurements of the Levels of Endogenous Opioid Peptides

The levels of M-ENK, *β*-EP, and DYN-A were measured through the method of SABC immunohistochemisty. The nuclei were identified according to the photographic atlas of the goat brain, and the morphological characteristics of the neurons [17–19].

**Figure 1:**
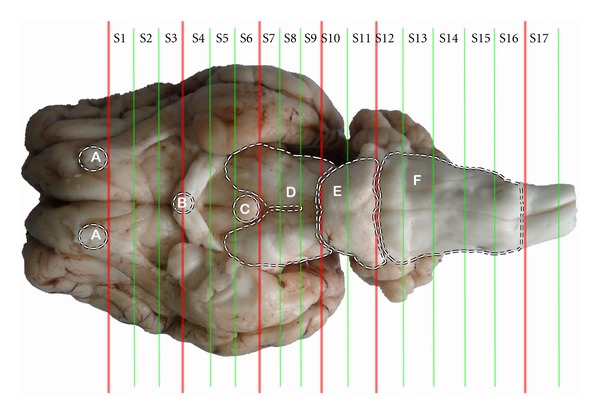
Brain sectionalization: (A) the residual part of the olfactory bulbs, (B) optic chiasm, (C) mamillary body, (D) cerebral peduncles, (E) Pons and (F) medulla oblongata. In the sections nuclei or areas: nucleus accumbens, septal area and caudate nucleus in S2, supraoptic nucleus, paraventricular nucleus of hypothalamus, and ventromedial nucleus of hypothalamus in S4, arcuate nucleus and amygdala in S5, habenular nucleus in S6, periaqueductal gray in S8, dorsal raphe nucleus and substantia nigra in S9, parabrachial nucleus and locus ceruleus in S10, nucleus raphe magnus in S13, solitary nucleus in S14, gigantocellular reticular nucleus in S15, and spinal cord dorsal horn in S17 are located.

Once the pain threshold was measured after EA, the goats were deeply anesthesized with intravenous administration of xylidinothiazoline at 3 mg/kg. Physiological saline was infused through bilateral carotid arteries at the same velocity with which the blood bled out from the jugular veins for about 5 min (until the blood fluid became colorless). Four percent paraformaldehyde instead of the physiological saline was infused for about 1 h. The brain and a part of the adjacent spinal cord were taken out of the skull and cervical vertebral canal. The brain was placed on a paraffin plate with the ventral surface up. Then it was transected into seven sections through the caudal edge of the residual part of the olfactory bulbs, the center of the optic chiasm, the caudal edge of the mamillary body, the sulcus between cerebral peduncles and pons, the sulcus between pons and medulla oblongata, and the caudal borderline between medulla oblongata and spinal cord, respectively. The first section with the residual part of the olfactory bulbs was discarded. The others were put into 4% paraformaldehyde to fix for 48 h. The cerebral cortex and cerebellum were stripped with the amygdala region left. Each of the second to fourth sections was evenly cut into three subsections (S1 to S9), while the fifth and the sixth sections were averagely divided into two and five subsections (S10 to S16), respectively. The seventh section was just spinal cord (S17). The sectionalization of the brain and the localization of nuclei and areas in subsections were illustrated in Figure 1. Each of the subsections was embedded in a paraffin block, sectioned at 5 *μ*m, mounted on polylysine-coated slides, deparaffinized, and rehydrated sequentially.

Twelve serial slides were chosen from near the middle of each subsection for immunohistochemical staining. Four of the twelve slides were randomly selected to detect the level of one of EOPs. Of these four slides, the three were incubated with one kind of rabbit-anti-M-ENK IgG (1 : 100), rabbit anti-*β*-endorphin IgG (1 : 200), or rabbit-anti-DYN-A IgG (1 : 100) (purchased from Wuhan Boster Biological Technology Ltd., Wuhan, China) while the rest was incubated with PBS instead of the corresponding antibody as negative control. Experimental procedures of SABC immunohistochemistry followed the instructions provided by the reagent company (Wuhan Boster Biological Technology Ltd., Wuhan, China). The cytoplasm of positive cells was stained as brown yellow. Optical density of the stained nuclei or area in the CNS was obtained with a light microscope connected to a video-based and computer-linked system (high-resolution pathological image analysis system-1000, Wuhan Qianping Ltd., Wuhan, China). This system was programmed to calculate the mean optical density (MOD) for three fields of each slide examined under 400× magnification. The level of EOPs in each nucleus or area was represented with the mean value ‰  of the mean optical density from the three slides.

### 2.5. Statistical Analysis

Statistical analysis was performed using SPSS version 18.0 (SPSS Inc., Chicago, IL, USA). All the data presented as mean ± SD. Pain threshold and EOP data were used for ANOVA followed by the Bonferroni's post hoc test. The correlation coefficient (Pearson's) was used to examine the relations between pain threshold and EOP level. Statistical significance was evaluated by determining if the *P* value was equal to or less than 0.05.

## 3. Results

### 3.1. Effects of EA of Different Frequencies on Pain Threshold

**Figure 2:**
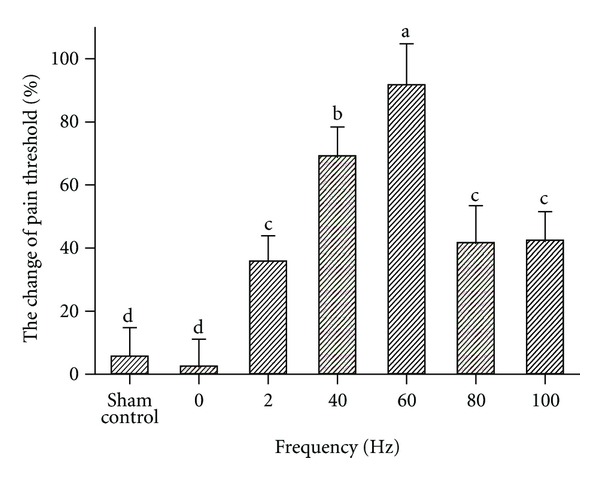
Pain threshold of goats stimulated by different frequencies (mean ± SD, %, *n* = 7). The same letter indicated that no significant difference in pain threshold between two frequencies (*P* > 0.05), and different letter indicated significant difference (*P* < 0.05).

The analgesic effects of EA of different frequencies in goats were expressed as the pain threshold (Figure 2). After EA treatment for 30 min, the pain threshold increased as frequency increased, reached the highest at 60 Hz, but decreased at 80 Hz. Frequencies of 100, 80, 60, 40, and 2 Hz increased pain threshold by 42%, 41%, 91%, 69%, and 35% (*P* < 0.01), respectively. The pain threshold of goats stimulated by 60 Hz was higher (*P* = 0.001) than that by 40 Hz. The pain threshold by either 60 Hz or 40 Hz was higher (*P* = 0.001) than that by 80, 100, or 2 Hz. The pain threshold between goats in sham control and 0 Hz was no difference (*P* = 1.000). Because there was no difference (*P* = 1.000) in pain threshold between goats stimulated with 80 and 100 Hz, the effect of 80 Hz on the release of EOPs was not considered in the following experiment.

### 3.2. Level of M-ENK Release Induced by Different Frequencies in the CNS

The levels of M-ENK were measured in the analgesia-related nuclei or areas which included nucleus accumbens (ACB), septal area (SA), caudate nucleus (CAU), amygdala (AMY), supraoptic nucleus (SON), paraventricular nucleus of hypothalamus (PVH), ventromedial nucleus of hypothalamus (VMH), periaqueductal gray (PAG), dorsal raphe nucleus (DR), substantia nigra (SN), parabrachial nucleus (PBN), locus ceruleus (LC), nucleus raphe magnus (NRM), and spinal cord dorsal horn (SCD). The release levels of M-ENK between the sham control and 0 Hz were no differences (*P* > 0.05) in the measured nuclei and areas. EA of different frequencies facilitated M-ENK release significantly (*P* < 0.05) in the measured nuclei or areas except NRM, SON and SCD (Table 1). 60 Hz induced M-ENK immunoactivities to increase by over 100% in the measured nuclei and areas except SN, SON, NRM, and SCD, and by over 300% in SA, AMY, and PAG. 100 Hz promoted M-ENK immunoactivities to increase by over 100% in SA, AMY, PVH, VMH, PAG, PBN, and LC, and by over 300% in PBN. 2 Hz increased M-ENK immunoactivities by over 100% in AMY, PVH and PAG. As frequency increased, M-ENK immunoactivities of the forebrain nuclei, and AMY, VMH, and PAG increased, reached the highest at 60 Hz, and then decreased at 100 Hz. There was no difference in M-ENK of VMH, SN or LC between goats stimulated by 60 Hz and 40 Hz, or by 60 Hz and 100 Hz. In DR, M-ENK immunoactivities elicited by 40 or 60 Hz were higher than those by 2 Hz or 100 Hz. There was no difference in M-ENK immunoactivities between goats stimulated by 40 Hz and 60 Hz in this nucleus. In PBN, M-ENK immunoactivities induced by 100 Hz were higher than those by 40 or 2 Hz (*P* = 0.0001), but not higher (*P* = 0.663) than those by 60 Hz. In SCD, 2 Hz caused M-ENK immunoactivities to increase (*P* = 0.0001) while the frequency of 40, 60 or 100 Hz did not. Statistic analysis showed that the pain thresholds correlated (*P* < 0.01) with M-ENK immunoactivities in the measured nuclei and areas except SON, NRM, and SCD. 

### 3.3. Level of *β*-EP Release Induced by Different Frequencies in the CNS

The *β*-EP levels were measured in the analgesia-related nuclei and areas which included ACB, SA, AMY, CAU, SON, arcuate nucleus (ARC), VMH, habenular nucleus (HB), PAG, DR, LC, PBN, NRM, solitary nucleus (SOL), and SCD. There were no differences (*P* > 0.05) in the *β*-EP immunoactivities between the sham control and 0 Hz in these nuclei and areas. 60 Hz increased *β*-EP immunoactivities by over 100% in the most measured nuclei and areas, and by over 300% in PAG and SOL, whereas 100 Hz increased *β*-EP immunoactivities by over 100% in ACB, VMH, PAG, LC, SOL, and AMY. 2 Hz increased *β*-EP immunoactivities by over 100% in PAG and SOL. Frequencies of 40, 60, and 100 Hz promoted *β*-EP immunoactivities to decrease (*P* < 0.05) in ARC, but to increase (*P* < 0.05) in the other measured nuclei or areas. The *β*-EP immunoactivities induced by 2 Hz were higher (*P* < 0.05) than those by 0 Hz in ACB, SA, SON, VMH, PAG, DR, LC, SCD, SOL, and AMY, but not in CAU, ARC, HB, PBN, and NRM. As frequency increased, EA promoted *β*-EP immunoactivities to change in the measured nuclei and areas except CAU, SON, ARC, HB, PBN, and AMY. In CAU, PBN, and AMY, *β*-EP immunoactivities induced by either 40 Hz or 60 Hz were higher (*P* < 0.05) than those by 2 Hz or 100 Hz. But no difference existed in *β*-EP immunoactivities between goats stimulated by 40 Hz and 60 Hz. In SON, there were no differences in *β*-EP immunoactivities between goats given with 60 Hz and 40 or 100 Hz. The pain thresholds correlated (*P* < 0.01) with *β*-EP immunoactivities in the measured nuclei and areas (Table 2).

### 3.4. Level of DYN-A Release Induced by Different Frequencies in the CNS

The levels of DYN-A were measured in CAU, SA, AMY, SON, PVH, VMH, PAG, PBN, gigantocellular reticular nucleus (GI), and SCD. In these nuclei and areas, DYN-A immunoactivities were no differences (*P* > 0.05) between the sham control and 0 Hz. DYN-A immunoactivities in the CNS increased in a frequency-dependent manner. Frequency of 40, 60, or 100 Hz promoted DYN-A to increase significantly (*P* < 0.05) in the CNS. The DYN-A immunoactivities induced by 100 Hz were different from those by 60 Hz in the measured nuclei and areas except VMH and GI. Statistic analysis showed that the pain thresholds correlated (*P* < 0.05) with DYN-A immunoactivities in the measured nuclei and areas except SCD (Table 3).

## 4. Discussion

### 4.1. The Measurement for Pain Thresholds and the Acupoint Selection for Electroacupuncture

There are a few methods to determine acupuncture-induced change in pain threshold. The tail flick response or paw withdrawal reflex by radiant heat can be used for the measurement of nociceptive threshold in rats [20, 21]. But it is not applicable for larger experimental animals (such as cattle and goats) because of their thick skin and hard hoof structure. The level of analgesia in these animals is commonly determined by scores based on an animal's response to a pinprick at a particular region [22, 23]. Obviously, this method is influenced by subjective factors. Ludbrook et al. [24] and Grant and Upton [25] measured the pain threshold in goats by using an algesimetry method based on a leg-lifting response to a subcutaneous electric stimulus. This method is not an involuntary reflex but instead a learned cognitive behavior. Additionally, it cannot be used for restrained animals. Potassium iontophoresis is a convenient and reliable experimental pain stimulus that can be presented rapidly and repeatedly with minimal loss in consistency of a subject's reported pain level [16]. In our study, potassium iontophoresis provided a tool for investigating changes in the pain thresholds of EA-treated goats.

A potent analgesic effect induced by EA depends on proper prescriptions of specific acupoints. “Zusanli” (St.36) and “Sanyinjiao” (SP.6) acupoints are commonly chosen for EA to elevate the pain threshold of the traumatic rats [26, 27]. A few sets of acupoints have been employed for EAA in ruminants. Numerous studies showed that EA at a set of Baihui, Santai, Ergen, and Sanyangluo acupoints elicited an effective analgesia in cattle [5]. Liu et al. [12] demonstrated that EA at this set of acupoints caused a potent analgesic effect in goats. In this study, we adopted this set of acupoints and obtained a similar analgesic effect as Liu did [12]. Experimental investigations showed that stimulation at different acupoints activated different nuclei and areas in rats [28–30]. However, whether acupoint specificities would change the releasing modalities of EOPs elicited by EA in the CNS of ruminants deserves to be investigated.

### 4.2. Distribution of Endogenous Opioid Peptides in the CNS of Goats

EOPs in the CNS include five families: enkephalins, endorphins, dynorphins, endomorphins, and orphanin FQ, of which the roles of M-ENK, *β*-EP and DYN-A in EA-induced analgesia were best studied. Studies showed that M-ENK was mainly found in CAU, hypothalamus, SON, PVH, VMH, midbrain, formatio reticularis mesencephali, SN, pons, and formatio reticularis medullae oblongatae [31, 32]. *β*-EP existed in hypothalamus, SON, ARC, parafascicular nucleus, preoptic region, interpeduncular nucleus, olfactory bulb, pons, medulla oblongata, SCD, AMY, cortex, and hippocampus [33–35]. DYN-A existed in SOL, medullary lateral reticular structure, preoptic area, periventricular nucleus, SON, ARC, SCD, hypothalamus, midbrain, and forebrain [36–38]. In the present study, higher level of *β*-EP was seen in ARC and SON, as had been reported elsewhere [34]. The rank order of DYN-A levels in our results was VMH > SON > PAG > GI = AMY = PBN = CAU = PVH > SCD, which was similar to reports in human and rats [39, 40]. The highest immunoactivities of M-ENK existed in SON, followed by the lower levels in ACB, CAU, PVH, SA, NRM, and SN, and the lowest levels in PAG, and AMY. These results were some different from the report by Shi et al. [41] that higher level of M-ENK existed in ACB, CAU, SON, PAG and AMY in rats. This discrepancy might be caused by species variation.

### 4.3. Different Frequencies Induced the Release Profile of EOPs in the CNS of Goats

The veterinary practice proved that frequencies of 40 to 100 Hz are believed to be proper for analgesia of ruminants [5]. But there is a lack of studies to specify this frequency range. In the present study, the increasing magnitude of the pain threshold in goats stimulated by 60 Hz was greater than that by the frequency of 100, 80, 40, or 2 Hz. Obviously, the analgesic effect by 60 Hz was better than that by the others. It is well documented that EOPs exhibit a frequency-dependent response in EA-produced analgesia in rats [42–44]. Low frequency (2 Hz) exerts antinociceptive effects mainly by enhancing the release of ENK and *β*-EP, whereas high frequency (100 Hz) produces antinociceptive effects by facilitating the release of DYN [44]. However, the release profile of goats' EOPs induced by different frequencies is not clear yet. In this study, 2 Hz and 100 Hz induced M-ENK to increase significantly in ACB, SA, CAU, AMY, PVH, PAG, DR, and LC and caused *β*-EP to increase significantly in ACB, SA, SON, VMH, PAG, DR, LC, SOL, and AMY. 60 Hz promoted the release of M-ENK or *β*-EP in the measured nuclei except in ARC. Therefore, 60 Hz activated more nuclei and areas to release M-ENK and *β*-EP than 2 or 100 Hz did in ruminants. EOPs participate in extensively physiological modulations. Their roles in EA-induced analgesia are verified by microinjecting EOP and its antagonist or antibody into some nuclei in rats. Levels of M-ENK in ACB [45], SA [46], CAU [47], PAG [48], or DR [48], AMY [49], and SN [50, 51] were proved to affect EA-induced analgesic effect. Either were the levels of *β*-EP in ACB [52], SA [46], CAU [53], PAG [54], DR [55], LC [56], NRM [56], HB [57], or ARC [58]. Our results showed that EA elevated the levels of M-ENK or *β*-EP in these nuclei of goats. Besides, we also found that M-ENK or *β*-EP immunoactivities increased in LC, PBN, VMH, SOL, SON, and PVH. It is seen that high frequencies can induce the simultaneous release of M-ENK or *β*-EP in a broader spectrum of nuclei in ruminants than in rats.

Role of DYN-A in EA-induced analgesia in the brain is controversial. Han and Xie [59] found that DYN-A did not produce EA-induced analgesia when it was microinjected into the cerebral ventricle of rats. Zhang et al. [60] made the opposite conclusion with DYN-A microinjection. In this study, EA induced DYN-A to increase in many analgesia-related nuclei in the CNS. The DYN-A immunoactivities induced by 100 Hz were significantly different from those by 60 Hz in the measured nuclei and areas except VMH and GI. It is shown that VMH and GI in the release of DYN-A were sensitive to both 100 Hz and 60 Hz. Whether the release of DYN-A takes part in EA analgesic modulation in the CNS of ruminants needs to be studied.

Release of DYN-A induced by 100 Hz in the SCD can produce a potent analgesic effect in rats [61]. In this study, EA of high frequencies induced DYN-A to increase in the SCD. This increase was in accordance with that of Han [61]. However, the increase in *β*-EP immunoactivities of the SCD and its correlation with the pain threshold values were different from the report of some studies in rats [62]. This discrepancy might be caused by the variation of the species or the studied spinal fragment. In this study, SCD samples were taken from the spinal cord adjacent to the medulla oblongata rather than the lumbar spinal cord.

Studies in rats showed that stimulation at 2 Hz and 100 Hz alternatively elicited the full release of M-ENK, *β*-EP, and DYN-A in the CNS, which produced a synergistic effect stronger than that at 2 Hz or 100 Hz alone [63]. Veterinary practice verifies that the mode of alternating stimulation with low and high frequencies can also induce more potent analgesic effect. However, the releasing modalities of EOPs which are induced by this stimulation mode in ruminants are worthy to be investigated.

### 4.4. Animal Species Variation of EA-Induced Analgesia

During the last decades, our understanding of how the brain processes acupuncture analgesia has undergone considerable development. But the major results of related researches are primarily obtained from small experimental animals such as rats, rabbits, dogs and monkeys. There are many factors which affect the EA-induced analgesic effect. Besides frequencies and acupoints, species-specificity has an important impact on EA analgesia. Studies showed that EA in combination with anesthetics led to reduce the dosage of the anesthetics in human, rat, and goat by 45%–55%, 50%–60%, and over 75%, respectively [12, 13]. Obviously, ruminants should be optimal model animals for research on the mechanisms of EA-induced analgesia. Our results showed that high frequencies motivated the simultaneous release of the three EOPs in the extensive analgesia-related nuclei and areas in the CNS, which may be conducive to explain why EA induced more potent analgesia in ruminants than in rats.

## 5. Conclusion

60 Hz was an optimal frequency for acupuncture-induced analgesia in goats and induced the simultaneous release of M-ENK, *β*-EP, and DYN-A in most of analgesia-related nuclei and areas in the CNS.

## Figures and Tables

**Table 1:** M-EMK immunoactivities induced by different frequencies in the CNS (mean ± SD, *n* = 7).

Nuclei and areas	Sham control	0 Hz	2 Hz	40 Hz	60 Hz	100 Hz	Correlation coefficients
ACB	15.60 ± 1.69^c^	15.30 ± 2.97^c^	24.56 ± 2.50^b^	32.69 ± 3.92^a^	35.15 ± 4.41^a^	22.59 ± 2.89^b^	0.888**
SA	15.14 ± 1.53^e^	14.77 ± 2.39^e^	25.05 ± 1.94^d^	54.95 ± 3.50^b^	60.51 ± 4.35^a^	33.81 ± 3.72^c^	0.923**
CAU	15.00 ± 1.92^c^	14.35 ± 3.18^c^	22.78 ± 2.92^b^	25.24 ± 5.64^b^	32.66 ± 3.47^a^	26.88 ± 5.48^b^	0.724**
AMY	10.47 ± 1.58^d^	10.04 ± 2.43^d^	20.49 ± 3.28^c^	35.06 ± 7.69^b^	47.28 ± 5.56^a^	38.45 ± 7.97a^b^	0.795**
SON	33.21 ± 2.58	32.99 ± 5.03	30.54 ± 6.16	31.68 ± 8.17	31.90 ± 6.49	33.73 ± 5.61	0.071
PVH	18.22 ± 3.59^b^	17.95 ± 4.25^c^	35.01 ± 6.18^b^	47.38 ± 7.63^a^	46.70 ± 10.22^a^	40.76 ± 3.55^ab^	0.734**
VMH	11.95 ± 1.72^b^	11.09 ± 3.29^b^	18.47 ± 2.56^b^	36.56 ± 8.99^a^	40.65 ± 10.10^a^	38.19 ± 9.27^a^	0.735**
PAG	8.05 ± 0.77^c^	7.75 ± 1.16^c^	20.53 ± 3.60^b^	23.29 ± 4.26^b^	33.71 ± 6.73^a^	16.74 ± 2.51^b^	0.808**
DR	11.56 ± 1.51^c^	11.34 ± 4.03^c^	16.73 ± 2.26^b^	29.01 ± 2.28^a^	26.30 ± 2.70^a^	20.49 ± 3.25^b^	0.837**
SN	12.72 ± 2.05^b^	12.42 ± 2.74^b^	15.14 ± 3.02^b^	22.95 ± 1.58^a^	22.64 ± 5.20^a^	20.36 ± 1.51^a^	0.721**
PBN	7.69 ± 1.04^c^	7.18 ± 1.61^c^	9.71 ± 1.52^c^	18.90 ± 2.93^b^	26.44 ± 3.53^a^	29.13 ± 3.01^a^	0.656**
LC	11.69 ± 1.15^c^	10.93 ± 0.89^c^	15.68 ± 2.97^b^	27.49 ± 2.11^a^	27.80 ± 3.60^a^	25.15 ± 3.31^a^	0.799**
NRM	14.15 ± 1.39	13.77 ± 2.74	15.77 ± 1.55	15.53 ± 3.92	17.07 ± 2.27	15.76 ± 4.28	0.263
SCD	5.22 ± 0.55^b^	5.22 ± 0.77^b^	7.31 ± 0.67^a^	6.00 ± 0.32^b^	5.85 ± 0.58^b^	5.44 ± 0.38^b^	0.123

ACB: nucleus accumbens, SA: septal area, CAU: caudate nucleus, AMY: amygdala, SON: supraoptic nucleus, SN: substantia nigra, PAG: periaqueductal gray, NRM: nucleus raphe magnus, PBN: parabrachial nucleus, LC: locus ceruleus, DR: dorsal raphe nucleus, SCD: spinal cord dorsal horn, PVH: paraventricular nucleus of hypothalamus, VMH: ventromedial nucleus of hypothalamus.

Note: There was difference (*P* < 0.05) between the values with different letters, and no difference (*P* > 0.05) with the same letters in a line. *means the levels of the endogenous opioid peptides correlate with the pain thresholds at the 0.05 level, and the levels at the 0.01 level. **The letters and symbols in the following tables have the same meanings as the table above.

**Table 2:** *β*-EP immunoactivities induced by different frequencies in the CNS (mean ± SD, *n* = 7).

Nuclei and areas	Sham control	0 Hz	2 Hz	40 Hz	60 Hz	100 Hz	Correlation coefficients
ACB	10.57 ± 1.48^d^	9.25 ± 0.44^d^	12.21 ± 2.00^c^	21.01 ± 1.80^b^	27.61 ± 1.91^a^	18.70 ± 1.21^b^	0.891**
CAU	20.90 ± 1.99^c^	20.84 ±3.87^c^	24.05 ± 2.99^bc^	36.60 ± 2.17^a^	36.99 ± 2.28^a^	26.81 ± 2.41^b^	0.835**
SA	21.04 ± 2.03^d^	20.65 ± 3.58^d^	29.58 ± 2.66^c^	42.08 ± 4.24^b^	49.52 ± 3.02^a^	31.25 ± 3.21^c^	0.893**
AMY	18.46 ± 2.45^d^	18.21 ± 3.33^d^	32.95 ± 4.16^c^	53.15 ± 2.74^ab^	54.59 ± 3.09^a^	48.86 ± 3.66^b^	0.840**
SON	29.31 ± 1.52^c^	28.88 ± 2.37^c^	38.18 ± 5.30^b^	53.13 ± 2.60^a^	52.68 ± 2.10^a^	51.25 ± 1.87^a^	0.783**
ARC	33.45 ± 2.11^a^	33.42 ± 2.34^a^	35.13 ± 2.02^a^	26.83 ± 1.13^bc^	23.76 ± 2.74^c^	28.83 ± 2.63^b^	0.795**
VMH	19.81 ± 2.10^e^	18.86 ± 1.79^e^	34.41 ± 2.82^d^	56.30 ± 3.77^b^	61.17 ± 2.35^a^	47.94 ± 1.66^c^	0.897**
HB	17.57 ± 2.42^c^	16.12 ± 1.63^c^	18.44 ± 1.49^c^	22.59 ± 2.05^b^	26.37 ± 1.20^a^	27.46 ± 1.30^a^	0.647**
PAG	8.40 ± 0.96^d^	8.22 ± 1.23^d^	19.04 ± 1.74^c^	28.82 ± 3.82^b^	33.70 ± 3.36^a^	30.18 ± 3.55^ab^	0.871**
DR	18.63 ± 1.83^d^	17.82 ± 2.34^d^	29.07 ± 1.94^c^	34.06 ± 3.09^ab^	38.43 ± 3.45^a^	31.10 ± 3.09^bc^	0.808**
LC	14.90 ± 2.21^d^	13.72 ± 2.95^d^	21.01 ± 2.37^c^	30.13 ± 2.66^b^	35.14 ± 2.42^a^	30.35 ± 1.80^b^	0.855**
PBN	7.65 ± 1.19^b^	7.54 ± 1.44^b^	8.69 ± 0.99^b^	14.07 ± 2.85^a^	14.92 ± 1.96^a^	7.99 ± 1.15^b^	0.790**
NRM	17.37 ± 2.20^c^	16.52 ± 2.23^c^	17.93 ± 1.21^c^	23.50 ± 1.65^b^	30.25 ± 2.91^a^	24.89 ± 1.76^b^	0.779**
SOL	11.50 ± 1.02^e^	11.35 ± 0.88^e^	23.41 ± 2.46^d^	33.83 ± 3.64^c^	49.46 ± 1.42^a^	43.59 ± 2.17^b^	0.928**
SCD	7.25 ± 1.13^d^	6.80 ± 0.26^d^	10.91 ± 1.00^c^	14.42 ± 1.18^b^	17.72 ± 1.03^a^	10.30 ± 1.26^c^	0.795**

ACB: nucleus accumbens, SA: septal area, CAU: caudate nucleus, AMY: amygdala, SON: supraoptic nucleus, ARC: arcuate nucleus, LC: locus ceruleus, PAG: periaqueductal gray, DR: dorsal raphe nucleus, PBN: parabrachial nucleus, HB: habenular nucleus, NRM: nucleus raphe, SOL: solitary nucleus, VMH: ventromedial nucleus of hypothalamus magnus, SCD: spinal cord dorsal horn.

**Table 3:** DYN-A immunoactivities induced by different frequencies in the CNS (mean ± SD, *n* = 7).

Nuclei and areas	Sham control	0 Hz	2 Hz	40 Hz	60 Hz	100 Hz	Correlation coefficients
CAU	9.03 ± 0.57^d^	8.89 ± 0.27^d^	9.77 ± 0.34^d^	17.22 ± 1.05^c^	20.91 ± 1.81^b^	25.37 ± 1.14^a^	0.573**
SA	14.93 ± 1.29^d^	14.68 ± 1.11^d^	14.88 ± 0.97^d^	19.82 ± 2.00^c^	26.61 ± 1.30^b^	35.75 ± 1.58^a^	0.400*
AMY	10.87 ± 1.10^e^	10.16 ± 0.65^e^	13.03 ± 1.55^d^	17.28 ± 1.40^c^	24.79 ± 1.42^b^	31.34 ± 1.44^a^	0.505**
SON	20.73 ± 1.52^c^	20.42 ± 1.08^c^	21.77 ± 1.41^c^	38.35 ± 2.51^b^	38.60 ± 0.94^b^	43.87 ± 1.77^a^	0.652**
PVH	8.74 ± 0.83^d^	8.65 ± 0.39^d^	8.85 ± 0.57^d^	17.53 ± 1.39^c^	19.87 ± 1.13^b^	23.24 ± 1.92^a^	0.606**
VMH	27.01 ± 1.15^c^	26.36 ± 1.10^c^	27.70 ± 1.22^c^	36.01 ± 1.83^b^	41.10 ± 1.89^a^	42.54 ± 1.49^a^	0.671**
PAG	12.27 ± 1.27^d^	12.02 ± 1.28^d^	12.87 ± 1.12^d^	16.94 ± 2.05^c^	19.68 ± 1.90^b^	23.72 ± 1.52^a^	0.457**
PBN	9.71 ± 0.74^d^	9.48 ± 0.55^d^	9.31 ± 0.50^d^	16.90 ± 1.73^c^	19.39 ± 1.44^b^	24.44 ± 1.19^a^	0.533**
GI	10.53 ± 1.01^c^	10.42 ± 1.32^c^	10.89 ± 1.58^c^	14.27 ± 1.85^b^	19.46 ± 1.60^a^	20.61 ± 1.34^a^	0.555**
SCD	3.73 ± 0.26^c^	3.68 ± 0.27^c^	4.06 ± 0.22^c^	5.86 ± 0.19^b^	6.05 ± 0.46^b^	10.27 ± 0.60^a^	0.275

CAU: caudate nucleus, SA: septal area, AMY: amygdala, SON: supraoptic nucleus, PAG: periaqueductal gray, PBN: parabrachial nucleus, GI: gigantocellular reticular nucleus, SCD: spinal cord dorsal horn, PVH: paraventricular nucleus of hypothalamus, VMH: ventromedial nucleus of hypothalamus.
